# Carbon benefits from Forest Transitions promoting biomass expansions and thickening

**DOI:** 10.1111/gcb.15292

**Published:** 2020-08-20

**Authors:** Pekka E. Kauppi, Philippe Ciais, Peter Högberg, Annika Nordin, Juha Lappi, Tomas Lundmark, Iddo K. Wernick

**Affiliations:** ^1^ Department of Forest Ecology and Management Swedish University of Agricultural Sciences (SLU) Umeå Sweden; ^2^ Department of Forest Sciences University of Helsinki Helsinki Finland; ^3^ Laboratoire des Sciences du Climat et de l’Environnement CEA‐CNRS UVSQ UPSACLAY Gif sur Yvette France; ^4^ Department of Forest Genetics and Plant Physiology Umeå Plant Science Centre Swedish University of Agricultural Sciences Umeå Sweden; ^5^ School of Forest Sciences Joensuu Finland; ^6^ Program for the Human Environment The Rockefeller University New York NY USA

**Keywords:** forest transitions, global carbon budget, missing sink, sustainable forestry, terrestrial ecosystems

## Abstract

The growth of the global terrestrial sink of carbon dioxide has puzzled scientists for decades. We propose that the role of land management practices—from intensive forestry to allowing passive afforestation of abandoned lands—have played a major role in the growth of the terrestrial carbon sink in the decades since the mid twentieth century. The Forest Transition, a historic transition from shrinking to expanding forests, and from sparser to denser forests, has seen an increase of biomass and carbon across large regions of the globe. We propose that the contribution of Forest Transitions to the terrestrial carbon sink has been underestimated. Because forest growth is slow and incremental, changes in the carbon density in forest biomass and soils often elude detection. Measurement technologies that rely on changes in two‐dimensional ground cover can miss changes in forest density. In contrast, changes from abrupt and total losses of biomass in land clearing, forest fires and clear cuts are easy to measure. Land management improves over time providing important present contributions and future potential to climate change mitigation. Appreciating the contributions of Forest Transitions to the sequestering of atmospheric carbon will enable its potential to aid in climate change mitigation.

## Graphical abstract

Abrupt losses of forest biomass are relatively easy to detect and monitor. Forestry reports are sometimes overly pessimistic as they acknowledge the biomass losses but fail to observe the gradual and subtle gains of biomass, which are difficult to detect empirically. Land management has promoted biomass gains at national and global levels.

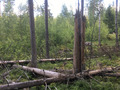

## 1 INTRODUCTION

Our aim in this paper is to complement the thoughtful analysis of Houghton (2020) on the global carbon cycle and echo his emphasis on the benefits of “understanding the potential for land management to remove CO_2_ from the atmosphere and understanding the processes responsible for the sink for carbon on land.” Here we draw attention to the role of land management in the ongoing growth and thickening of the terrestrial carbon stock. “Forest Transition” refers to a turn‐around from shrinking to expanding forests as experienced by many nations in the previous centuries (Mather, 1992; Meyfroidt & Lambin, 2011). Forest Transitions have contributed to the increasing global tree cover since 1982 and have become common on all continents, save Africa and South America, where they remain the exception rather than the rule (Song et al., 2018). Initially, Forest Transition referred to *land area* as the landscape attribute (Mather, 1992). More recently, it has been applied also to forest *biomass density* as a shift from degrading to becoming denser (Kauppi et al., 2006; Le Noë et al., 2020).

Forests emit carbon dioxide when they shrink or degrade. Conversely, forests show net uptake of carbon dioxide when they expand as stocks of carbon in vegetation and soils increase. Even if forest area remains constant, its carbon density (kg carbon per square meter) can change. The cumulative stock change since 1957 in the northern hemisphere is estimated at +78 PgC including both vegetation and soils (Ciais et al., 2019). Assuming 70% realized in biomass (Pan et al., 2011) this means nearly a doubling of forest biomass since 1957, a huge increase in terrestrial biomass in so short a time.

Forest Transitions accelerate the gross removal of carbon from the atmosphere and decelerate gross emissions of terrestrial carbon. In addition, Forest Transitions improve global opportunities for obtaining sustainable timber, which can displace more energy intensive materials in the economy and reduce the use of marginal forestry lands for roundwood harvests, considered to be critically important for long‐term climate change mitigation (Smith et al., 2014).

## 2 FOREST TRANSITION OF AREA AND DENSITY: CASE NATIONS

The transition from shrinking to expanding forests began in industrialized nations in the middle of the 19th century. In the decades since 1950, often without significant changes in forest area, forest biomass density has increased. The relative importance of density increase versus area expansion varies in time and space as these country abstracts demonstrate.

### 2.1 France

A first documentation of Forest Transition was published for France showing a turn‐around from shrinking to expanding forest area in the early 19th century (Mather, Fairbairn, & Needle, 1999). More recently, Le Noë et al. (2020) showed that during the last 50 years the carbon stock of forests in France has grown from increases in biomass density. They also discussed how trees serve as a pathway for carbon from the atmosphere into soils. The stock of carbon in forest soil has correlated positively with the stock of carbon in the tree cover.

### 2.2 European Russia, Ukraine and Belarus

A Forest Transition has been documented in easternmost Europe as agriculture declined after the dissolution of the Soviet Union. From 1990 to 2009 an estimated 31 million ha of cropland was abandoned in European Russia, Ukraine and Belarus (Schierhorn et al., 2013). Most of the abandoned lands have sprouted sparse young forests that act as emerging carbon sinks. These lands represent an important reserve for global food production (Meyfroidt, Schierhorn, Prishchepov, Müller, & Kuemmerle, 2016).

### 2.3 East Asia

Forest expansion in East Asia has been most pronounced in China and Japan (Fang et al., 2014). A combination of factors served to release marginal lands to forest expansion including the national tree planting program, the migration to the cities, the intensification of agriculture, and the opening of the Chinese food sector to imports. Between 1990 and 2015, China’s forest area increased from 157 to 208 Mha, and woody biomass changed from 8.5 to 12.8 billion tons C according to official statistics (FAO, 2015, ref. China). Hence, the rate of expansion was 32% for forest area and 50% for forest biomass, suggesting that significant densification is going on in China’s forests.

### 2.4 Ireland

Since 2000 industrial tree plantations continue to expand in many countries including Ireland. Trees chosen for industrial plantations are selected to grow fast. Globally, plantation forests cover about 131 million ha, which is 3% of the global forest area and 45% of the total area of planted forests (FAO, 2020). In Ireland, from 2005 to 2015, forests expanded by 8% in terms of forest area and 68% in their growing stock volume (FAO, 2015, ref. Ireland), indicating a much faster rate of growth in biomass than in area.

## 3 ABRUPT LOSSES, SUBTLE GAINS

Biomass losses are often abrupt, sudden and obvious (Körner, 2003) to the noticeable exception of degradation of tropical forests. Large losses such as clear cuts, large‐scale insect defoliations and wild‐fires are easy to detect using remote sensing methods. In contrast, biomass gains that contribute to carbon sequestration are gradual, subtle, and difficult to detect. Once a forest canopy is established, biomass accumulates below as incremental thin layers of wood cells covering the trees’ trunk, a process difficult to quantify. Monitoring carbon gains in soils is even harder. Remote sensing may not resolve the incremental changes from in the steady accrual of terrestrial carbon, although recent advancement of the research methods show certain promise (Fan et al., 2019; Wigneron et al., 2020). Subtle net gains of carbon at stand level are best observed empirically using the FLUXNET system (Baldocchi et al., 2001) which detects CO_2_ exchange at the stand level but provides an insufficient basis for global extrapolations. The global network monitors carbon gains in growing young and middle aged forests excluding the unpredictable locations where stand‐replacing disturbances happen to occur (Körner, 2003).

We expand the criticism by Hansen, Potapov, and Tyukavina (2019) to Baccini et al. (2017) by drawing attention to an evident measurement bias which has led to underestimation of subtle biomass gains. Baccini et al. (2017) estimate that tropical forest vegetation lost carbon in 2003–2014. Their method however was sensitive to the abrupt losses, but not the subtle gains. Methodologically, they estimated for each pixel a piecewise linear function for the changes of carbon. They then tested separately for each pixel whether the function deviates statistically significantly (with *p* < .05) from a no‐change, intercept‐only model.

Baccini et al. (2017) assumed that a given pixel that did not indicate a statistically significant change meant there indeed was no change. This criterion excluded a great majority (79%) of the pixels from the analysis, because no significant change was detected. The carbon sink/source of tropical forests was computed from the remaining pixels, in which the stock change was either positive (6%) or negative (15%). In spite of the apparent plausibility, the argument is untenable. If one tested simultaneously the hypothesis that in all of the excluded pixels there was zero change, this hypothesis would become rejected. There are good reasons to assume that most of the no‐change pixels show, owing to regular forest growth, a slight positive change. This example demonstrates that subtle changes of forest growth and densification may have remained obscured at regional, national and global levels.

## 4 THE CONCENTRATION OF AGRICULTURE AND FORESTRY

A growing literature attempts to describe the long‐term mechanisms driving Forest Transitions (Rudel et al., 2020). Changes in farming technologies have allowed farmlands around the globe to go fallow. Relocating agriculture from marginal lands to more productive fields relaxes the demand of land for food production allowing marginal agricultural lands to return to forests. Improving farm yields have spared large areas of land from food production (Ausubel, Wernick, & Waggoner, 2013). Mather and Needle (1998) emphasized the dynamic effects of concentrating crop cultivation and animal husbandry on the best, most productive lands. State of the art agricultural technologies are most rewarding economically when implemented on the best farmlands. The best soils globally are still used for agriculture, but agriculture has abandoned some of the less productive agricultural soils, which then have become excellent forest soils. This mechanism offers potential to have a significant impact moderating or reducing global land for the rest of the century (Folberth et al., 2020). Modern food industries, packaging and retail have succeeded in controlling food losses, also reducing the need for land, even though much remains to be improved in this sector (Bajželj et al., 2014).

Changes in forest management and forest products industries have complemented the changes in the food sector in driving the terrestrial sequestration of carbon. Net increases in forest extent occur by means of spontaneous forest expansion, active planting, or both. Because of forest management, modern forests grow faster than their predecessors (Henttonen, Nöjd, & Mäkinen, 2017; Pretzsch, 2009). Due to improvements in international transportation, forest products such as pulp can reach international markets easily. This has allowed pulpwood farms to be planted on the most productive forest soils, thus sparing marginal timber lands from harvesting and allowing them to be conserved for nature. Improved industrial processes and recycling have reduced demand for virgin material from the forest (Wernick, Waggoner, & Ausubel, 2000). Other developments reducing demand for forest products include electronic communication replacing print media and reduced use of fire‐wood and charcoal in the economy.

## 5 CARBON SINK AND FOREST TRANSITIONS

Managed forests cover about three quarters of the global forests (Potapov et al., 2008). Sustainable forest management can lead to increases in forest extent as well as forest density, providing key elements for mitigating climate change in the decades ahead. Understanding Forest Transitions contributes to explaining the bias of underestimating the role of land management as a driver of the terrestrial carbon sink (Erb et al., 2013). To date, Forest Transitions have greatly served to sequester many tons of carbon dioxide and prevented carbon from remaining in the atmosphere.

The terrestrial sink has not only been large in the global carbon budget but growing in proportion to emissions. During 1959–2012, approximately 350 billion tonnes of carbon were emitted by humans to the atmosphere, of which about 55% was taken up by the land and oceans (Ballantyne, Alden, Miller, Tans, & White, 2012). For 2009–2018, the terrestrial sink (“gross gain”) was estimated at 3.2 ± 0.6 PgC/year while the terrestrial emissions (“gross loss”) were estimated at 1.5 ± 0.7 PgC/year (Friedlingstein et al., 2019). We propose that Forest Transitions played a growing role, not yet fully appreciated in accelerating the carbon sink in terrestrial ecosystems. Across the large areas of global lands where Forest Transition has occurred (Meyfroidt & Lambin, 2011), vegetation biomass carbon builds up responding to an expansion of both forest area and forest density (Le Noë et al., 2020).

Bookkeeping models track losses and gains of forest area and the regrowth of secondary forests. They use annual rates of land‐use change (ha/year) to describe per hectare changes in carbon stocks. Rates of forest regrowth and rates of decomposition of organic debris are fixed in bookkeeping models. As a result they do not capture possible changes that influence increases in total carbon sequestration in recent decades. Because landowners and governments invest in forests with the explicit goal of accelerating stand establishment and promoting forest growth, managed forests grow—and sequester more carbon—faster.

We contend that only by neglecting the effects of the accumulated carbon due to Forest Transitions do Houghton and Nassikas (2018) estimate gross emissions and removals of carbon from forests from land management as +5.5 and −4.4 PgC/year, respectively, yielding net emissions of +1.1 PgC/year globally. (Positive signs refer to emissions to the atmosphere, negative to removals from the atmosphere). These estimates only partially account for subtle processes including forest densification and carbon accumulation in forest soils. Accounting for these processes, that may go undetected, yields stronger gross sink than −4.4 PgC/year if impacts of land management and Forest Transitions are fully acknowledged. We do not challenge Houghton’s (2020) estimate of the terrestrial net sink, −1.7 PgC/year, but we attribute more of the sink to land management as opposed to environmental change. Our view supports government policies that encourage improved land management, more effective forest growth and better use of agricultural and wood products. The enhancement of carbon sequestration through more focused forest management are not restricted the potential new forest, established forests can also sequester more carbon in the future.

To promote both carbon sequestration in forest ecosystems and the renewable timber available from managed forests, governments can encourage forest management by implementing legislation and incentives. Rudel et al. (2020) suggest that government policies are becoming an important driver of new Forest Transitions in the 21st century. Public policy action directly promotes tree planting for the promotion of carbon sequestration, such as the campaign to plant “trillion trees” (Trilloin Tree Campaign, 2020). In 2020, it remains too early to observe the results from recent policy initiatives in the ground data. Over time, we expect that drivers of Forest Transitions will evolve due to changes in forests and in parallel to societal changes in economic development, urbanization and globalization.

We agree with Houghton (2020) that attribution of carbon fluxes to management, as opposed to environmental change, is difficult in practice. The global environment has changed and affected forest growth especially in the boreal region (Kauppi, Posch, & Pirinen, 2014). The sporadic thickening observed in the most northern Russian forests (Forbes, Fauria, & Zetterberg, 2010), is not related to changes in land use but changes in ambient environmental conditions. Nevertheless, our perception on the important role of Forest Transitions has policy implications. Methods of agriculture and practices of both growing and using round wood can be improved. Positive impacts of management can extend to all forests, not only to the potential new forests, which eventually become created (Bastin et al., 2019). Forest Transitions have dual impacts. They affect both carbon sequestration in forest ecosystems and the potential of renewable timber available from managed forests.

## 6 FOREST TRANSITIONS AND RENEWABLE CARBON

The core challenge of the global climate policy is the reduction of emissions from the combustion of fossil carbon. Vast quantities of coal and oil are consumed annually. Global forest growth cannot fulfil the diverse and large demands of modern energy consumption (Smil, 2017). Trying to replace the combustion of coal and oil by biomass for energy production is thus untenable as a standard solution (Chatham House, 2017). This said, Forest Transitions add to the potential of obtaining renewable wood for human consumption.

We support Houghton (2020) in encouraging Earth system models (global dynamic global vegetation models) to incorporate changes of land management in the models. These models calculate departures from preindustrial fluxes of carbon by differencing two simulations, one with climate and CO_2_ held constant since 1700, and one with the changes in these two environmental drivers. Land management has dramatically changed since 1700 from predominantly intense deforestation for agricultural land, wood harvest, cultivation of soil, to eventual improving yields, reforestation, tree planting, and active fire management. These changes have affected Forest Transitions, which have constituted a positive anthropogenic effect on the global carbon cycle.

## 7 CONCLUSIONS

Forest Transitions have played an important role, which has not yet been fully acknowledged and quantified, in promoting the acceleration of the carbon sink in terrestrial ecosystems. Neither bookkeeping models nor global land models as elements of earth system models acknowledge the full effect of Forest Transitions on the carbon sink of terrestrial ecosystems. Complex evolution in agricultural and forestry technologies, urbanization, globalization, consumption patterns, and public policies affect changes of vegetation and soils on global lands. Forest Transition theory describes not only the direction, but also attempts to quantify the rate of change as forest carbon stock and offers additional new insight for improving the estimates of the global terrestrial carbon sink. Including the effects of Forest Transitions especially on changes of carbon density in vegetation and soils can contribute to improved understanding of the global carbon cycle.

Terrestrial ecosystems are likely to sequester carbon effectively in the coming decades, owing to the strong and persistent trend of forests around the globe transitioning to become larger and denser. One important question is: What will be the fate of forest biomass in the long term? When can we expect carbon sequestration to cease and when will we see forests naturally entering a period of being neither net removers nor net contributors of carbon to the atmosphere? Europe was the first continent to experience Forest Transitions, and Europe’s forest biomass has shown first signs of saturation (Nabuurs et al., 2013). The mechanism is clear: gross uptake of carbon will equal gross removals of carbon through respiration, harvests and mortality as the forest carbon stock approaches a steady state under the specific management regime (regardless of forest harvests or no harvests). This appears inevitable in the long‐term future. However, young growing forests in many other areas of the world have decades of carbon absorption ahead. At present, the net effect of global terrestrial ecosystems is positive in the global carbon budget sequestering more than 10% of the fossil emissions while, nevertheless, provisioning food, lumber and fiber products to the growing population. Building up the stocks of carbon through incremental Forest Transitions is a mid‐term instrument in climate policy, while sustainable provisioning of food and biomass with constantly improving methods is an open‐ended future goal of environmental policy, into the 22nd century and beyond.

## CONFLICT OF INTEREST

None.
